# Cell surface engineering via self-assembly DNA networks for cell behavior control

**DOI:** 10.3389/fbioe.2026.1834218

**Published:** 2026-05-13

**Authors:** Tianshu Chen, Xuexue Liu, Xiaochen Tang, Ji Ma, Siwei Mao, Miao Ding, Ni Zhen, Qizhi Diao, Qiuhui Pan

**Affiliations:** 1 Department of Clinical Laboratory, Hainan Branch, Shanghai Children’s Medical Center, School of Medicine, Shanghai Jiao Tong University, Sanya, China; 2 Clinical Laboratory, Shanghai Children’s Medical Center, School of Medicine, Shanghai Jiao Tong University, Shanghai, China; 3 School of Pharmacy, Faculty of Medicine, Macau University of Science and Technology, Macau, China

**Keywords:** cell migration and invasion, cell surface engineering, DNA networks, extracellular matrix mimic, integrin

## Abstract

Synthesis of the extracellular matrix provides a novel opportunity to cell behavior control. However, exploration of the application of nucleic acid materials for cell surface engineering to achieve physical regulation of migration remains relatively limited. In this study, a self-assembled DNA network anchored to the cell membrane was constructed as a synthetic matrix mimic, which has been shown to inhibit cancer cell migration and invasion through physical confinement. The DNA network forms a dense and uniform engineering on the surface of tumor cells through a process of self-assembly. This study found that coating with RCA1/2 network led to a significant reduction in wound healing rate of approximately 49.19% and invasion rate of approximately 44.49%. The study also indicates that the potential mechanism for inhibiting cell migration and invasion is that coating of the DNA network on the membrane significantly limits membrane fluidity and promotes integrin retention on the membrane, thus interfering with its dynamic circulation. The spatial confinement in this study provides a novel DNA-based platform for controlling cell behavior as well as opening up a new paradigm for anti-migratory and anti-invasive strategies.

## 1 Introduction

Precise control of cell behavior represents a significant area of research in the disciplines of synthetic biology and regenerative medicine (Niu et al., 2017). However, the most challenging problem in cancer treatment is its dysregulation, especially the abnormal acquisition of cancer cell migration and invasion ability during tumor metastasis (Condeelis and Pollard, 2006). Tumor metastasis is recognized as the most significant challenge in cancer therapy, accounting for approximately 90% of cancer-related mortality. Tumor metastasis involves a complex cascade of events that includes cancer cell detachment from the primary tumor, invasion of the extracellular matrix, intravasation into the circulatory system, and colonization of distant organs (Méndez et al., 2018). Although targeted therapy has made some progresses, single pathway blockade strategies frequently prove ineffective due to tumor heterogeneity (Lee et al., 2018; Zhou M. et al., 2024). Therefore, development of strategies to simultaneously regulate multiple metastatic pathways has become imperative to overcome therapeutic limitations. In this context, precise engineering of the cell surface, which serves as an interface for cell-cell interactions and the microenvironment, provides a novel approach for the inhibition of tumor metastasis.

Membrane fluidity serves as the core physical basis that enables cancer cells to acquire invasive motility (Zhang et al., 2025). Current research has indicated that metastatic tumor cells exhibit a significant increase in membrane fluidity compared with primary tumor cells. This increase in membrane liquidity has been shown to accelerate key transfer processes such as integrin clustering, growth factor receptor activation, and exosome secretion (Sherbet, 1989). However, current methods of regulation have significant limitations. Traditional membrane fluidity regulators (e.g., cholesterol analogues) lack cell specificity and are capable of interfering with normal cell function (Ivankov et al., 2023). In addition, static surface engineering is unable to respond to the dynamic changes in the tumor microenvironment, which complicates the achievement of precise inhibition with high efficiency and low toxicity (Abbina et al., 2018; Zhou et al., 2025). Consequently, the development of a cell surface engineering strategy that combines targeting, dynamic response capability, and good biocompatibility is of significant scientific importance and clinical translational value for cell behavior control in the inhibition of tumor metastasis.

The emergence of DNA nanotechnology has provided a new toolkit for engineering cell surfaces. Due to the sequence programmability, structural designability, and excellent biocompatibility, DNA have the ability to form various structures through self-assembly (Wang et al., 2024). Li et al. have developed a DNA-directed molecular polymerization reaction for single-cell encapsulation: a pair of DNA primers is first introduced onto the cell surface, followed by rolling-circle amplification (RCA) directly on the cell membrane; the two resulting RCA products then interweave with each other to form a cocoon-like structure that encapsulates the cell for subsequent analysis (Gao et al., 2019). When bioorthogonal chemistry technology is employed in conjunction with DNA, it can be covalently anchored to the cell membrane, thereby enhancing its stability (Chen et al., 2024; Chen et al., 2021). Here, a self-assembled DNA network was developed on the surface of cancer cells to inhibit migration and invasion through physical confinement. It has been demonstrated that this DNA network can significantly reduce cell migration and invasion rates, with the mechanism being closely related to membrane fluidity limitation and integrin membrane retention. This study proposes a novel strategy for physical regulation that does not depend on particular signaling molecules. It provides innovative and universal solutions for anti-migratory and anti-invasive strategies and opens up new directions for the application of synthetic extracellular matrix mimic in tumor therapy.

## 2 Materials and methods

### 2.1 Cell culture

The human hepatoblastoma cells (HepG2 cells), human cervical cancer cells (HeLa cells), human breast cancer cells (MDA-MB-231 cells) were obtained from the Institute of Biochemistry and Cell Biology (Chinese Academy of Science). Cells were cultured in DMEM supplemented with 10% FBS at 37 °C in a humidified atmosphere containing 5% CO_2_. The medium was refreshed every 24 h. Cells were passaged using trypsin upon reaching confluence, and those in the logarithmic growth phase were harvested for subsequent experiments.

### 2.2 Synthesis of rolling circle amplification (RCA) products

To synthesize RCA1, 500 nM Primer 1 was hybridized with 100 nM CircDNA 1, followed by ligation with 100 U T4 DNA ligase in ligation buffer (1 mM ATP) at 37 °C for 1 h. Subsequently, 4 U phi29 DNA polymerase, 200 μM dNTPs, and 200 μg/mL BSA were added to initiate amplification at 37 °C for 4 h. The reaction was terminated via incubation at 95 °C for 10 min to yield long-chain RCA1. RCA2 was synthesized following the same protocol by replacing Primer 1/CircDNA 1 with Primer 2/CircDNA 2.

### 2.3 Characterization of RCA products

RCA products were analyzed using 0.6% agarose gel electrophoresis. Samples (5 μL) were mixed with 1 μL of 5× loading buffer, loaded onto the gel, and electrophoresed in 1× TAE buffer at 120 V for 30 min. For atomic force microscopy (AFM) characterization, RCA1 and RCA2 were deposited onto freshly cleaved mica surfaces and dried in the dark. Images were acquired in tapping mode at a scan rate of 0.3 Hz, with a probe frequency ranging from 160 to 260 kHz. Molecular beacon (MB) binding was validated by incubating 500 nM 1 or beacon 2 with their respective RCA templates at 37 °C for 0.5 h. Fluorescence spectra were recorded (λ ex/em = 550/570 nm for Cy3-MB 1, 650/670 nm for Cy5-MB 2).

### 2.4 Surface assembly of DNA networks on engineered cells

Cells were first treated with 50 μM Ac_4_ManNAz for 72 h to introduce azido groups. After washing with DPBS, 2 μM DBCO-DNA was added and incubated at 37 °C for 0.5 h to yield DNA-functionalized cells via click chemistry. To assemble the network, 100 μL of RCA1 and RCA2 were added to the cells (either sequentially or as a pre-mixed 1:1 ratio) and incubated at 37 °C for 0.5 h. For visualization, cells were incubated with 500 nM beacon 1 and beacon 2 for 0.5 h, and then fixed with 4% PFA, stained with DAPI, and imaged via confocal microscopy.

### 2.5 Cell migration and invasion assays

For wound healing assays, DNA-engineered cells were seeded in 12-well plates and treated with RCA products. A scratch was introduced using a sterile pipette tip, and the cells were cultured in serum-free medium for 24 h. The wound area was measured using ImageJ software. The wound healing rate was calculated as (initial wound area–final wound area)/initial wound area × 100%. The reduction in wound healing was calculated as (healing rate_control_–healing rate_treated_)/healing rate_control_ × 100%. For invasion assays, cells were seeded into Matrigel-coated Transwell upper chambers in 1% FBS medium, while the lower chambers contained 10% FBS medium. After 24 h, cells were fixed with 4% PFA, stained with 0.1% crystal violet, and imaged. The invasion rate was calculated as (number of invading cells_treated/number of invading cells_control) × 100%. The reduction in invasion was calculated as (invasion rate_control_ − invasion rate_treated_)/invasion rate_control_ × 100%.

### 2.6 Cytotoxicity and apoptosis analysis

Cell viability and proliferation were assessed using the CCK-8 assay. DNA-engineered HepG2 cells were seeded in 96-well plates and incubated with RCA1 and RCA2 for 0.5 h at 37 °C. Absorbance at 450 nm was measured after 4 h of incubation with the CCK-8 reagent at various time points (12–72 h). For apoptosis analysis, cells were processed using the PI/Annexin V-FITC kit according to the manufacturer’s instructions and analyzed by flow cytometry.

### 2.7 Statistical analysis

Two-tailed Student’s t-test was used for evaluating statistical mean differences between two groups. All statistical analyses were performed using the GraphPad software (Prism 7), and *p* < 0.05 was regraded statistically significant.

Detailed experimental procedures, and sequencel used in this study are provided in the Supplementary Material.

## 3 Results

**FIGURE 1:**
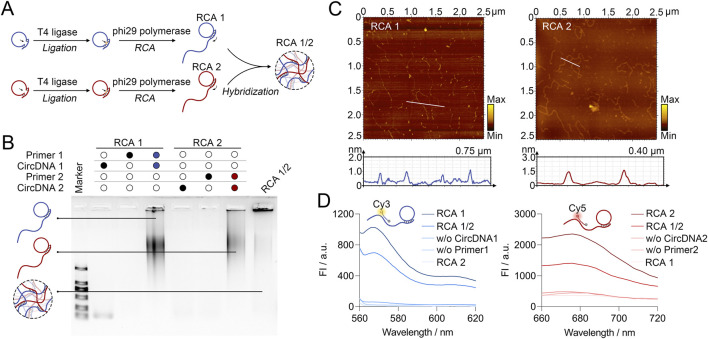
**(A)** Schematic illustration of the preparation of the networked RCA1/2 by mixing two long-chain DNA products (RCA 1 and RCA 2). **(B)** Agarose gel electrophoresis of the formation of products synthesized by RCA and the assembly of RCA1/2. **(C)** Atomic force microscopy (AFM) images of RCA 1 and RCA 2. **(D)** Fluorescence analysis of the combination of RCA1 to molecular beacon 1, RCA2 to molecular beacon 2, respectively. *n* = 3 independent experiments.

The DNA network bound to the cell surface is assembled through the complementary hybridization of two long-chain DNA products synthesized by rolling circle amplification (RCA). Firstly, two long-chain DNA products, designated as RCA1 and RCA2, were synthesized and characterized. The principle is as shown in Figure 1A. The split circular DNA (CircDNA) was first complementary to the primer DNA (Primer) and then formed a closed circular DNA in the presence of T4 ligase. Next, RCA reaction was initiated with the CircDNA as the template, in the presence of Primer and phi29 polymerase, to synthesize long-chain DNA products (RCA1 or RCA2). RCA1 and RCA2 could be further assembled to form a networked DNA structure (RCA1/2) by base complementary pairing. Agarose gel electrophoresis was used to analyze RCA1, RCA2, and RCA1/2. As shown in Figure 1B, the successful synthesis of RCA1 resulted in the observation of diffuse bands in Lane 3, whereas no visible band was observed in the absence of either Primer (lane 1) or CircDNA (lane 2). The electrophoresis results of RCA2 were similar to those of RCA1 (lane 4–6). The bands within the gel wells indicates the formation of high-molecular-weight RCA products, consistent with previous reports (Dean et al., 2001; Hanif et al., 2023; Zhou Q. et al., 2024). The molecular weight of the band (lane 7), which was produced by mixing RCA1 and RCA2, was significantly higher than that of RCA1 or RCA2 alone. These results indicate that RCA1 and RCA2 can be combined to form a networked RCA1/2 with a higher molecular weight.

Next, the morphology of RCA products was characterized by atomic force microscopy (AFM). As shown in Figure 1C, both RCA1 and RCA2 are characterized by long-chain DNA structures, thereby confirming the success of RCA. Subsequently, two groups of molecular beacons were designed for cell surface imaging of RCA1/2. The beacon 1 was labeled with a Cy3 fluorophore at the 5′ end and a quencher at the 3′ end, which was used for tracing RCA1. The beacon 2 was labeled with a Cy5 fluorophore at the 5′ end and a quencher at the 3′ end, which was employed for RCA2. In order to explore the specific binding ability of RCA1 and RCA2 to beacons, we performed a fluorescence intensity analysis. As shown in Figure 1D, the presence of RCA1 resulted in a significant increase in beacon 1 fluorescence intensity. Conversely, in the absence of RCA1 or the presence of RCA2 alone, low fluorescence intensity was detected. Similarly, an increase in beacon 2 fluorescence intensity could be detected only in the presence of RCA2. These results demonstrate the feasibility that RCA1 and RCA2 can be synthesized successfully, fluorescently traced using beacon 1 and beacon 2, respectively, as well as self-assembling to form RCA1/2.

**FIGURE 2:**
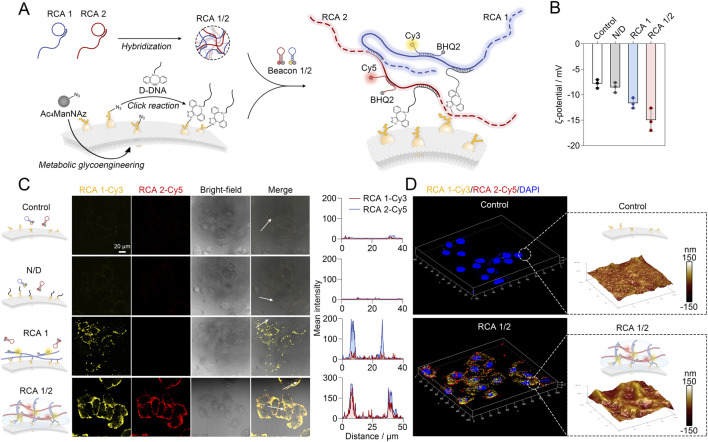
**(A)** Schematic illustration of the D-DNA engineering of HepG2 cells and the self-assembly of RCA1/2 network on the surface of cells. **(B)** Zeta potential of HepG2 cells after the D-DNA engineering, RCA1 binding alone and RCA1/2 network coating, respectively. Data are the means ± SD, *n* = 3 independent experiments. **(C)** Confocal fluorescence images and co-localized analysis of HepG2 cells after the D-DNA engineering, RCA1 binding alone and RCA1/2 network coating, respectively. Scale bar = 20 μm. **(D)** The three-dimensional fluorescence and atomic force microscopy (AFM) images of HepG2 cells after the RCA1/2 network coating. The nucleus was stained with DAPI. Cy3-labdled molecular beacon 1 and cy5-labeled molecular beacon 2 were adopted to image the RCA1 and RCA2, respectively.

Next, a networked RCA1/2 was constructed on the surface of DNA-engineered human hepatoblastoma cells (HepG2 cells) by DNA self-assembly using long-chain RCA1 and RCA2, and was visualized using MB1 and MB2 (Figure 2A). In brief, the cells were first treated with tetra-acylated N-azidoacetylmannosamine-tetraacylated (Ac_4_ManNAz), followed by incubation with dibenzocyclooctyne (DBCO)-functionalized DNA strands (D-DNA) to obtain D-DNA engineered HepG2 cells via bioorthogonal chemistry. Then RCA1 and RCA2 were introduced, which could not only hybridize with D-DNA on the cell surface, but also cross-link with each other, thus forming an interconnected RCA1/2 on the cell surface. Zeta potential analysis shows that D-DNA engineering, RCA1 binding alone and RCA1/2 network coating result in a gradual increase in the negative charge on the cell surface, which is consistent with the negative charge characteristics of DNA (Figure 2B). It is worth noting that cells coating the RCA1/2 network exhibited the highest negative charge, indicating maximal DNA loading on the cell surface. In order to visualize RCA1/2 on the surface of D-DNA engineered HepG2 cells, Cy3-labeled MB1 and Cy5-labeled MB2 were employed to detect RCA1 and RCA2, respectively. As shown in Figure 2C, no observable fluorescence signal was detected in the control group and the D-DNA engineered cells that did not coat to RCA1/2 (N/D group). The cells that only exhibited a combination with RCA1 displayed strong Cy3 fluorescence (RCA1 group). Meanwhile, the cells that combined with RCA1/2 network exhibited Cy3 and Cy5 signals simultaneously, while these signals were clearly co-localized, distributed evenly, and densely located on the cell surface (RCA1/2 group). Flow cytometry analysis further confirmed substantial DNA binding on the cell membrane, with up to 83.08% of cells exhibiting RCA1/2 network combination (Supplementary Figure S1). In order to investigate the accuracy of the results, t is necessary to determine whether Förster resonance energy transfer (FRET) occurs between Cy3 and Cy5 after RCA1/2 assembly. As shown in Supplementary Figure S2, the calculated FRET efficiency was a lowly 3.80%, confirming the absence of FRET between Cy3 and Cy5 in the RCA1/2 network. Similar results were obtained by three-dimensional fluorescence imaging and AFM analysis on the cell membrane (Figure 2D), thereby demonstrating the formation of a network-like structure by RCA1/2 on the cell surface. In terms of stability, the covalent anchoring via bioorthogonal DBCO-azide chemistry ensures robust attachment of the DNA network to the cell membrane, resisting rapid internalization and degradation, as evidenced by sustained fluorescence signals over 12 h (Supplementary Figure S3). The absence of cytoplasmic fluorescence at all time points indicates that the RCA1/2 network remains predominantly surface-bound and undergoes minimal internalization during the 36 h observation period. These results indicate that the self-assembled RCA1/2 network has potential application prospects for the simulation of extracellular matrix.

**FIGURE 3:**
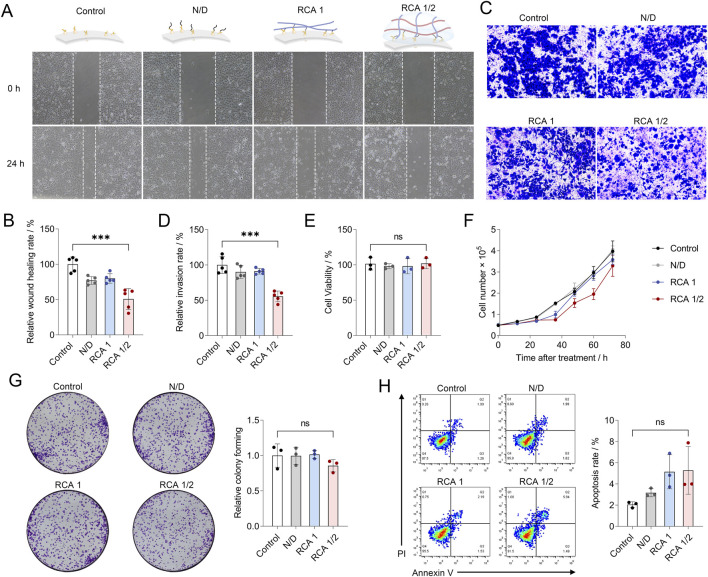
**(A)** Wound healing assay of the effect of RCA1/2 network on the migration of HepG2 cells. **(B)** Statistical results of the relative wound healing rate obtained from **(A)**. Data are the means ± SD, *n* = 5 independent experiments. **(C)** Transwell matrigel invasion assay of the effect of RCA1/2 network on the invasion of HepG2 cells. **(D)** Statistical results of the relative invasion rate obtained from **(C)**. Data are the means ± SD, *n* = 5 independent experiments. **(E)** Cell viability analysis of HepG2 cells after RCA1/2 network coating by CCK-8 assays. Data are the means ± SD, *n* = 3 independent experiments. **(F)** Cell proliferation analysis of HepG2 cells after RCA1/2 network coating by CCK-8 assays. Data are the means ± SD, *n* = 3 independent experiments. **(G)** The proliferation ability of HepG2 cells after RCA1/2 network coating by clone formation assays. Data are the means ± SD, *n* = 3 independent experiments. **(H)** Cell apoptosis of HepG2 cells after RCA1/2 network coating by flow cytometry. Data are the means ± SD, *n* = 3 independent experiments. *p* values were calculated by the Student’s t-test: *p < 0.05, **p < 0.01, ***p < 0.001.

Next, the effect of the RCA1/2 network as a synthetic extracellular matrix mimic for cell behavior control was investigated. We first explored the migration and invasion of HepG2 cells after the RCA1/2 network coating. As shown in Figures 3A,B, the results of wound healing assay show that coating with the RCA1/2 network was significantly reduced the wound healing rate of HepG2 cells. Meanwhile, D-DNA engineering or RCA1 binding alone exhibited no significant effect on cell migration. Quantitative analysis revealed that compared with the untreated control group, the wound healing rate of the HepG2 cells after the RCA1/2 network coating decreased by approximately 49.19% (*p* ≤ 0.001). Subsequently, the transwell matrigel invasion assay was performed to explore the effect of coating with the RCA1/2 network on cell invasion. As shown in Figures 3C,D, the number of invasive cells in the RCA1/2 group was greatly reduced compared to untreated control group, D-DNA engineering group and RCA1 binding group. Quantitative analysis showed that compared with the control group, the invasion rate of the RCA1/2 group decreased by approximately 44.49% (*p* ≤ 0.001). To investigate the generalizability of our findings, we applied the RCA1/2 network to human cervical cancer cells (HeLa cells) and human breast cancer cells (MDA-MB-231 cells). As shown in Supplementary Figures S4, S5, the RCA1/2 network effectively inhibited migration by 37.98% in HeLa cells and by 44.23% in MDA-MB-231 cells, while significantly reducing invasion by 41.74% in HeLa cells and by 47.65% in MDA-MB-231 cells. These results are similar to those observed in HepG2 cells, indicating that the inhibitory effect of the RCA1/2 network is broadly applicable across different cancer cell lines. These results indicate that the self-assembled RCA1/2 network on the cell membrane can inhibits cell migration and invasion. It is possible that this inhibitory effect is attributable to the dense network structure formed on the cell surface. The abundant DNA strands on the surface of engineered cells may promote the close complementary pairing between RCA1 and RCA2, forming a denser RCA1/2 network. The DNA network physically blocks the interaction between cells and the extracellular matrix, thereby affecting cell migration and invasion. Furthermore, a scrambled DNA sequence that lacks complementarity to the D-DNA on the cell surface was introduced. This scrambled DNA did not appreciably affect cell migration or invasion, confirming that the inhibitory effect is specific to the assembled RCA1/2 network rather than a non-specific consequence of DNA coating (Supplementary Figure S6).

Subsequently, the effects of the RCA1/2 network as a synthetic extracellular matrix mimic on cell viability, proliferation and apoptosis were explored. Cell viability analysis was first performed by cell counting kit-8 (CCK-8) assay. Results showed that in comparison with the untreated control group, the cell viability was not significantly influenced after the coating of the RCA1/2 network on the cell membrane (Figure 3E). In addition, D-DNA engineering or RCA1 binding alone had no significant impact on cell viability. Similar results were obtained in the cell proliferation experiment: there was no significant difference in cell proliferation within 72 h between the DNA engineering, RCA1 alone binding and RCA1/2 network coating groups and the untreated control group (Figure 3F). The *in vitro* clone formation assays provided further confirmation that coating with the RCA1/2 network on the cell membrane had no significant effect on the clone formation ability (Figure 3G). Cell apoptosis was then explored by flow cytometry. Results showed that compared with the untreated control group, the apoptosis rate of DNA engineering, RCA1 alone binding and RCA1/2 network coating groups did not change significantly (Figure 3H). Thees results may be attributable to the low-toxicity and biocompatibility of DNA as a biomaterial.

**FIGURE 4:**
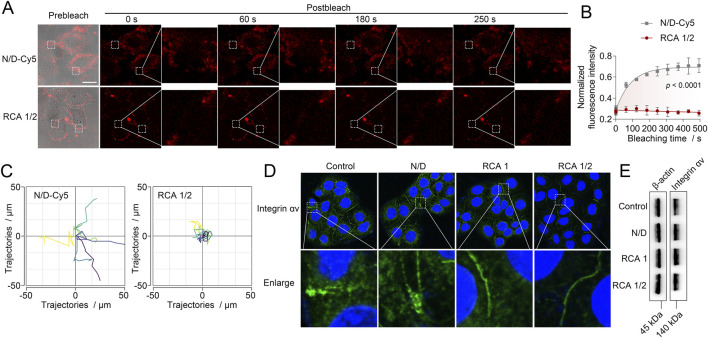
**(A)** Fluorescence recovery after photobleaching (FRAP) analysis of HepG2 cells after the D-DNA-Cy5 engineering and RCA1/2 network coating. Scale bar = 20 μm. **(B)** Plots of fluorescence intensity in the box region in **(A)** versus time after photobleaching. Data are the means ± SD, *n* = 3 independent experiments. **(C)** Single-particle tracking maps of fluorescent dots in the box region in **(A)**. **(D)** Confocal fluorescence images of integrin αv on HepG2 cells after the D-DNA engineering, RCA1 binding alone and RCA1/2 network coating, respectively. **(E)** Western blotting results of integrin αv in HepG2 cells after the D-DNA engineering, RCA1 binding alone and RCA1/2 network coating, respectively.

Finally, we investigated the potential mechanisms by which the RCA1/2 network inhibits cell migration and invasion. Since the RCA1/2 network has been observed to form a dense structure on the cell surface, and since cell migration and invasion processes are highly dependent on dynamic membrane remodeling, it can be hypothesized that the inhibition of cell migration and invasion by the RCA1/2 network may be related to changes in cell membrane fluidity (Taraboletti et al., 1989). Membrane fluidity was assessed using fluorescence recovery after photobleaching (FRAP) and single-particle tracking, both of which provide sufficient sensitivity and spatial resolution to detect changes in membrane dynamics. The effect of RCA1/2 network coating on HepG2 cell membrane fluidity was first investigated using the FRAP. We replaced D-DNA with D-DNA labeled with Cy5 for imaging of D-DNA-Cy5 engineered cells, and used beacon2-Cy5 for imaging of cells coated with RCA1/2 network. As shown in Figures 4A,B, in D-DNA-Cy5 engineered HepG2 cells, the fluorescence on the membrane rapidly recovered after photobleaching, indicating high membrane fluidity. In contrast, HepG2 cells coated with the RCA1/2 network exhibited minimal fluorescence recovery, with the signal remaining quenched after photobleaching. Single-particle tracking of fluorescent dots on the membrane showed that, compared with D-DNA-Cy5 engineered cells, the RCA1/2 network-coated cells displayed significantly reduced trajectories and diffusion coefficients, indicating that membrane fluidity was significantly constrained (Figure 4C).

The process of cell migration and invasion is closely related to the dynamic distribution of integrins. Integrins, as the primary cell adhesion transmembrane receptors, sense microenvironmental signals and regulate cell morphology through the extracellular endocytosis cycle. As the principal transmembrane adhesion receptors, integrins are capable of receiving microenvironmental signals and regulating cell morphology (Kechagia et al., 2019; Pang et al., 2023). During the process of cell migration, integrins undergo dynamic transportation between the intracellular and membrane compartments. This process is dependent on membrane fluidity and deformability. We therefore investigated the effect coating with the RCA1/2 network on the localization of cellular integrins. Immunofluorescence staining experiments showed that, in comparison with untreated control group, D-DNA engineering group and RCA1 binding group, HepG2 cells coated with RCA1/2 network exhibited a significant enrichment of integrins in the cell membrane components and a reduction in cytoplasmic localization (Figure 4D). At the same time, Western blotting results showed that there was no significant change in the total expression of integrins in HepG2 cells after coating with the RCA1/2 network (Figure 4E). These results indicate that the RCA1/2 network alters the subcellular distribution of integrins, promoting their retention on the cell membrane. To investigate the integrin trafficking, we performed an antibody internalization assay to quantify anti-integrin antibody internalization using flow cytometry. Cells from four groups—untreated control, D-DNA engineered, RCA1 alone, and RCA1/2 network—were incubated with a fluorescently labeled anti-integrin antibody at 4 °C to label surface integrins. Then, the cells were incubated at 37 °C to allow internalization. After stripping the surface-bound antibodies with acid, we measured the internalized fluorescence. As shown in Supplementary Figure S7, the RCA1/2 network group exhibited a significantly lower anti-integrin antibody internalization rate than the untreated control, D-DNA-engineered, and RCA1 alone groups (*p* < 0.01). These results demonstrate that the RCA1/2 network impairs integrin endocytic trafficking, leading to integrin retention on the plasma membrane.

Based on the results obtained from Figure 4, it can be concluded that the inhibitory effect of the RCA1/2 network on cell migration and invasion may be achieved through the following mechanisms: firstly, a dense RCA1/2 network forms a physical barrier on the cell surface, limiting the lateral diffusion of membrane lipids and proteins, thereby reducing membrane fluidity; secondly, the decrease in membrane fluidity interferes with the dynamic circulation of integrins, causing them to abnormally reside on the membrane, which may weaken integrin-mediated adhesion renewal and signal transduction, ultimately inhibiting cell migration and invasion ability.

## 4 Discussion

In this study, we successfully constructed a self-assembled DNA network (RCA1/2) based on rolling circle amplification systematically investigated the effects and potential mechanisms as a synthetic extracellular matrix mimic for cell behavior control. Firstly, the successful synthesis of two long-stranded DNA strands, RCA1 and RCA2, was confirmed, as well as their complementary self-assembly ability. Subsequently, bioorthogonal chemistry was employed to anchor DNA onto the cell membrane, thereby allowing for the formation of a uniform and dense RCA1/2 network on the surface of DNA engineered HepG2 cells. Functional assays demonstrated that coating with RCA1/2 network led to a significant inhibition of the migration and invasion of DNA-engineered HepG2 cells, with a reduction in wound healing rate of approximately 49.19% and invasion rate of approximately 44.49%. Finally, mechanism research has indicated that this inhibitory effect may be associated with the regulation of cell membrane fluidity by the RCA1/2 network. FRAP experiments and single particle tracking have confirmed that the coverage of the RCA1/2 network significantly limits the lateral diffusion of membrane lipids and proteins, leading to a significant decrease in membrane fluidity. Meanwhile, the subcellular distribution of integrins was altered with an enrichment from the cytoplasm to the membrane, while their total expression level remained unaltered.

A comparison of DNA self-assembly strategy with existing cell surface engineering approaches is summarized in Supplementary Table S2. Notably, the proposed method in this work exhibits high efficiency (approximately 83.08% cell coverage) with uniform network formation, offers potential reversibility through nuclease treatment or strand displacement, demonstrates excellent biocompatibility as evidenced by viability and proliferation assays, and provides a unique mechanism of action via physical confinement and integrin retention, distinguishing it from chemical- or polymer-based systems. It should be noted that while results offer significant support for a causal relationship between reduced membrane fluidity, impaired integrin trafficking, and suppressed cell migration and invasion, the specific downstream signaling pathways—such as those involving focal adhesion kinase (FAK), Rho GTPases, or actomyosin contractility—remain to be fully elucidated. This represents a current limitation of our study, which we plan to address in future investigations. Besides, while it cannot be formally ruled out that the DNA coating may nonspecifically affect other membrane proteins, control experiments with non-assembled (RCA1 alone) or scrambled DNA coatings—which introduced comparable surface charge and steric hindrance—did not impair migration or invasion, arguing against a dominant nonspecific effect. Subsequent studies employing surface proteomics or single-molecule tracking could systematically evaluate potential off-target effects on other receptors or ion channels. In addition, the effects of RCA1/2 network on non-cancerous cells remain to be evaluated in future studies. From a translational perspective, the network effectively suppresses migration and invasion across multiple cell lines and its physical mechanism independent of specific mutations suggest potential as a broad-spectrum anti-metastatic coating or cell-surface therapeutic platform.

In summary, this study reveals a synthetic extracellular matrix mimic based on DNA self-assembly that can regulate cell behavior through a dual mechanism of physical barriers and molecular retention, providing a new strategy for intervening in tumor cell migration and invasion, and also providing a theoretical basis for the design and biomedical applications of artificial extracellular matrix.

## Data Availability

The original contributions presented in the study are included in the article/Supplementary Material, further inquiries can be directed to the corresponding authors.
