# Supplementary material for: Cell surface engineering via self-assembly DNA networks for cell behavior control
Source: Front Bioeng Biotechnol. 2026 May 13;14:1834218. doi: 10.3389/fbioe.2026.1834218 (PMC13212446; doi:10.3389/fbioe.2026.1834218)
Supplement: Supplementary file 1 [file Supplementaryfile1.docx]

Supplementary Material

# Experimental Section

**Materials and Reagents.** All DNA oligonucleotides were synthesized by Sangon Biotech. Co., Ltd (sequences listed in Table S1). (Shanghai, China)with HPLC purification. Tetraacetylated N-azidoacetylmannosamine (Ac_4_ManNAz) was purchased from Click Chemistry Tools LLC (Scottsdale, USA). T4 DNA ligase and phi29 DNA polymerase were obtained from New England Biolabs (NEB). 5× loading buffer and Dulbecco's phosphate-buffered saline (DPBS) were sourced from Shanghai Jierui Bioengineering and Shanghai Sangon Biotech, respectively. 4% paraformaldehyde (PFA) and crystal violet staining solution were purchased from Shanghai Sangon Biotech. 4',6-Diamidino-2-phenylindole (DAPI), Cell Counting Kit-8 (CCK-8), and the PI/Annexin V-FITC apoptosis detection kit were obtained from Jiangsu Kaiji Biotechnology. Dulbecco’s Modified Eagle Medium (DMEM) and fetal bovine serum (FBS) were purchased from Gibco. All antibodies were purchased from Abcam. Matrigel and Transwell chambers were obtained from BD Biosciences and Corning, respectively. Ultrapure water was produced using a Milli-Q system.

Confocal imaging was performed using a Zeiss LSM 710 laser scanning confocal microscope. Gel imaging was conducted with a Bio-Rad GelDoc XR system. Flow cytometry was carried out using a Beckman Coulter CytoFLEX. Absorbance and fluorescence measurements were recorded using a Molecular Devices SpectraMax M2 microplate reader and a Hitachi F-7100 fluorescence spectrophotometer, respectively.


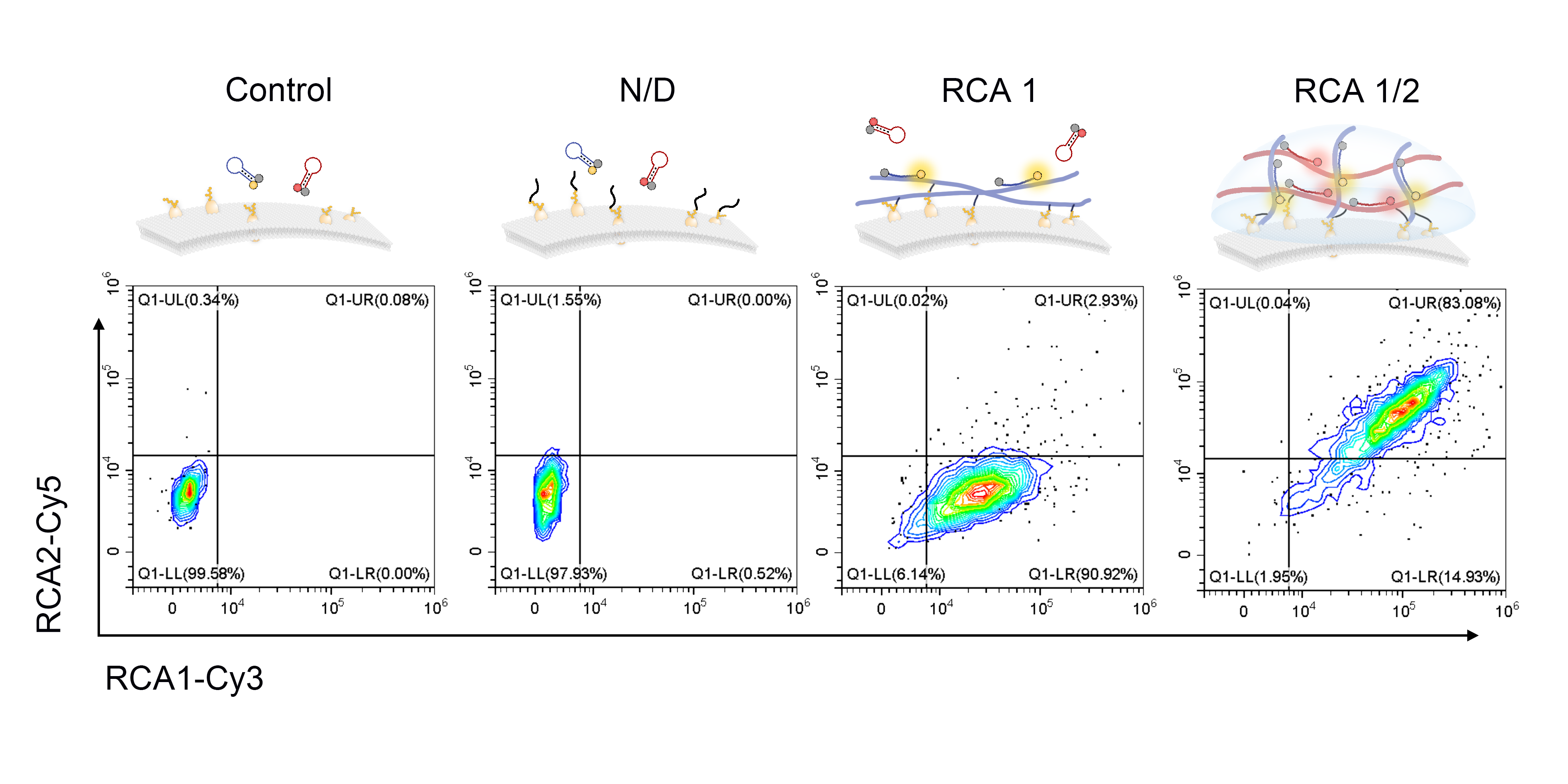


Figure S1 Flow cytometry analysis of HepG2 cells after the D-DNA engineering, RCA1 binding alone and RCA1/2 network coating, respectively.


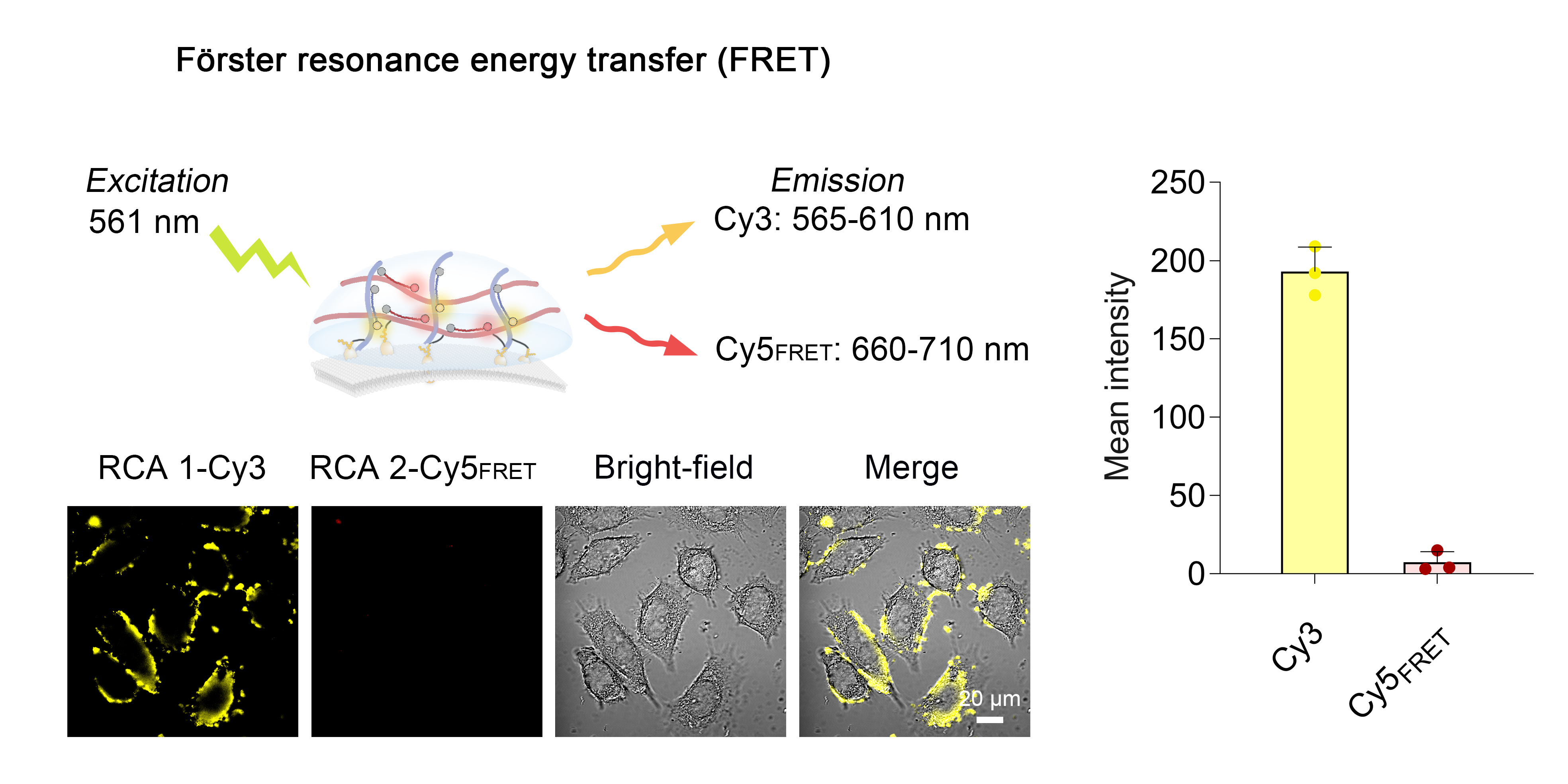


Figure S2 Förster resonance energy transfer (FRET) analysis between Cy3 (donor) and Cy5 (acceptor) after RCA1/2 assembly. Cells coated with RCA 1/2 network were labeled with Cy3-MB1 and Cy5-MB2. FRET analysis was performed on a confocal microscope using a 561 nm laser at 100% power. FRET efficiency was calculated as E=(Intensity_acceptor_ / Intensity_donor_) × 100%. The calculated FRET efficiency was 3.80%. Data are the means ± SD, *n*=3 independent experiments.


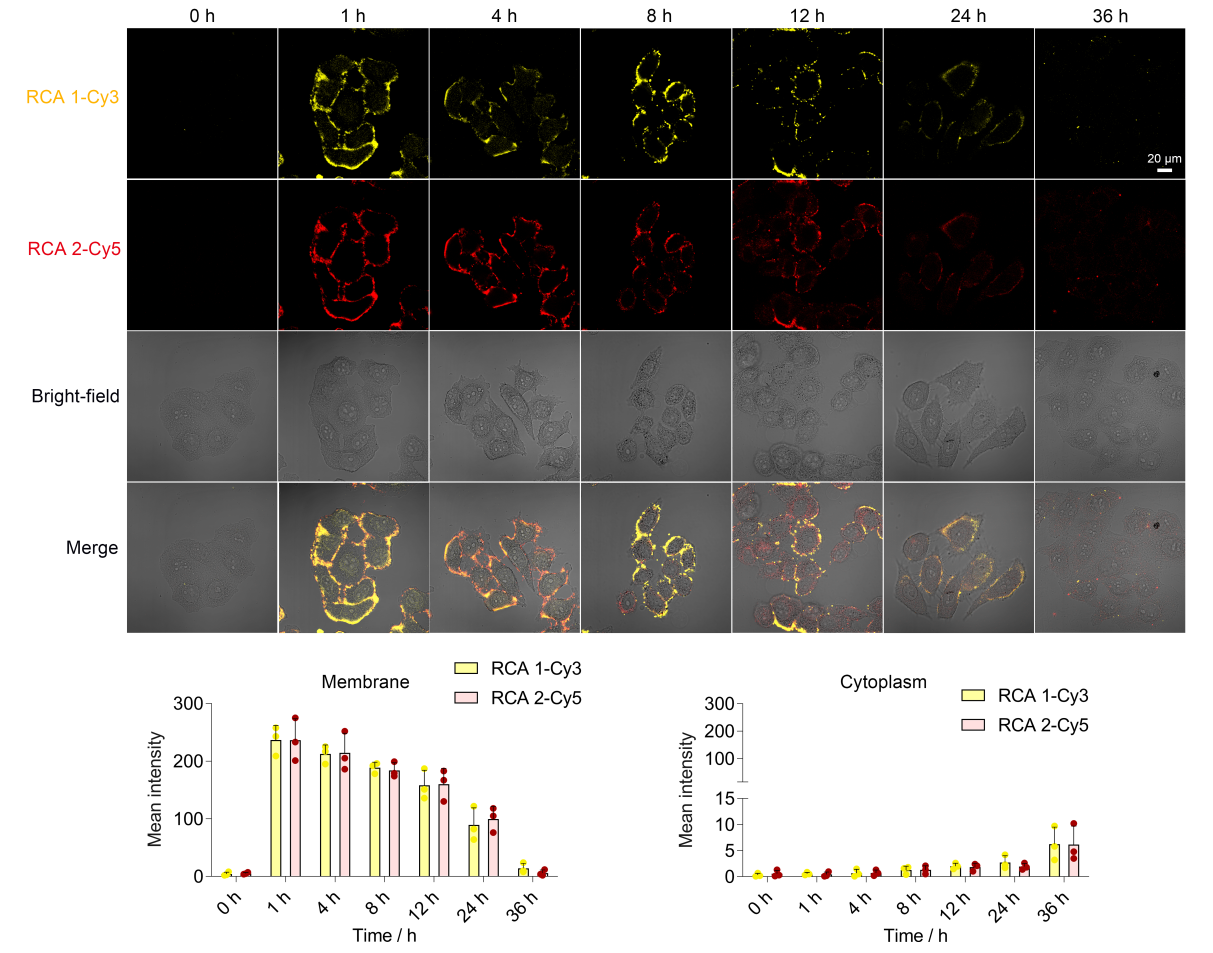


Figure S3 Confocal fluorescence images and mean fluorescence intensity analysis of D-DNA engineered HepG2 cells after the RCA1/2 network coating for varied durations. Scale bar = 20 μm. Data are the means ± SD, *n*=3 independent experiments.


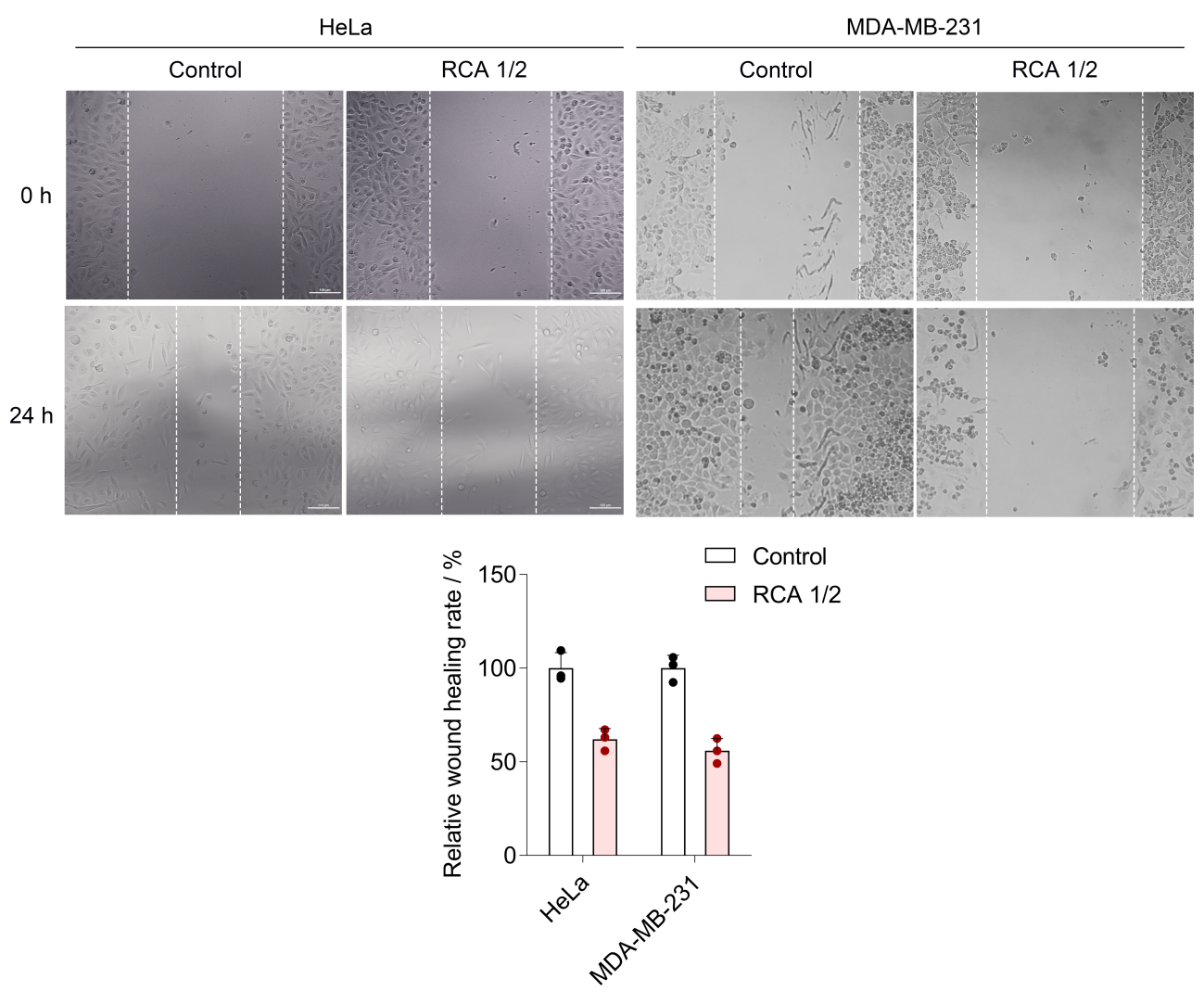


Figure S4 Wound healing assay and statistical results of the effect of RCA1/2 network on the migration of HeLa and MDA-MB-231 cells. Data are the means ± SD, *n*=3 independent experiments.


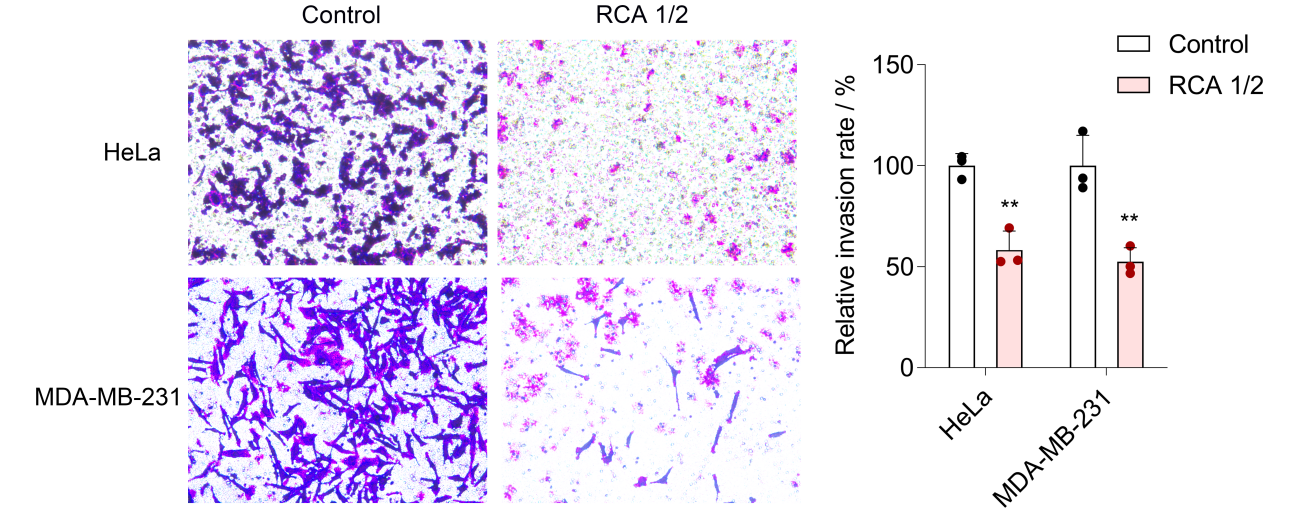


Figure S5 Transwell matrigel invasion assay and statistical results of the effect of RCA1/2 network on the invasion of HeLa and MDA-MB-231 cells. Data are the means ± SD, *n*=3 independent experiments.


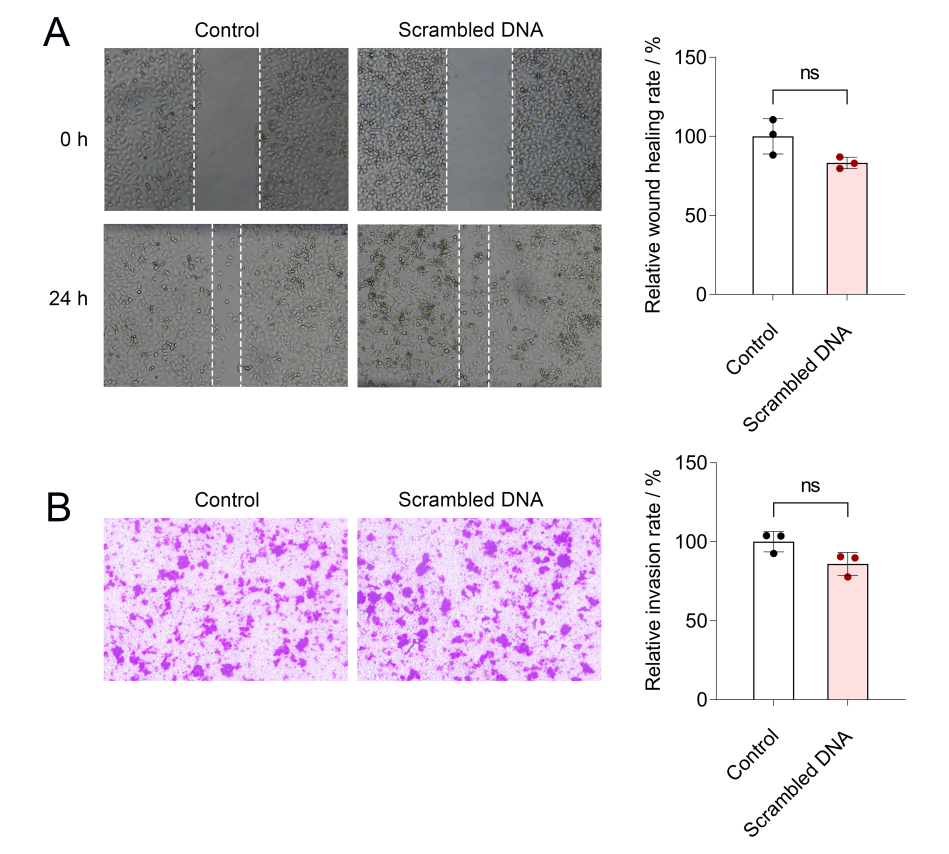


Figure S6 (A) Wound healing assay and statistical results of the effect of scrambled DNA on the migration of HepG2 cells. (B) Transwell matrigel invasion assay and statistical results of the effect of scrambled DNA on the invasion of HepG2 cells. Data are the means ± SD, *n*=3 independent experiments.


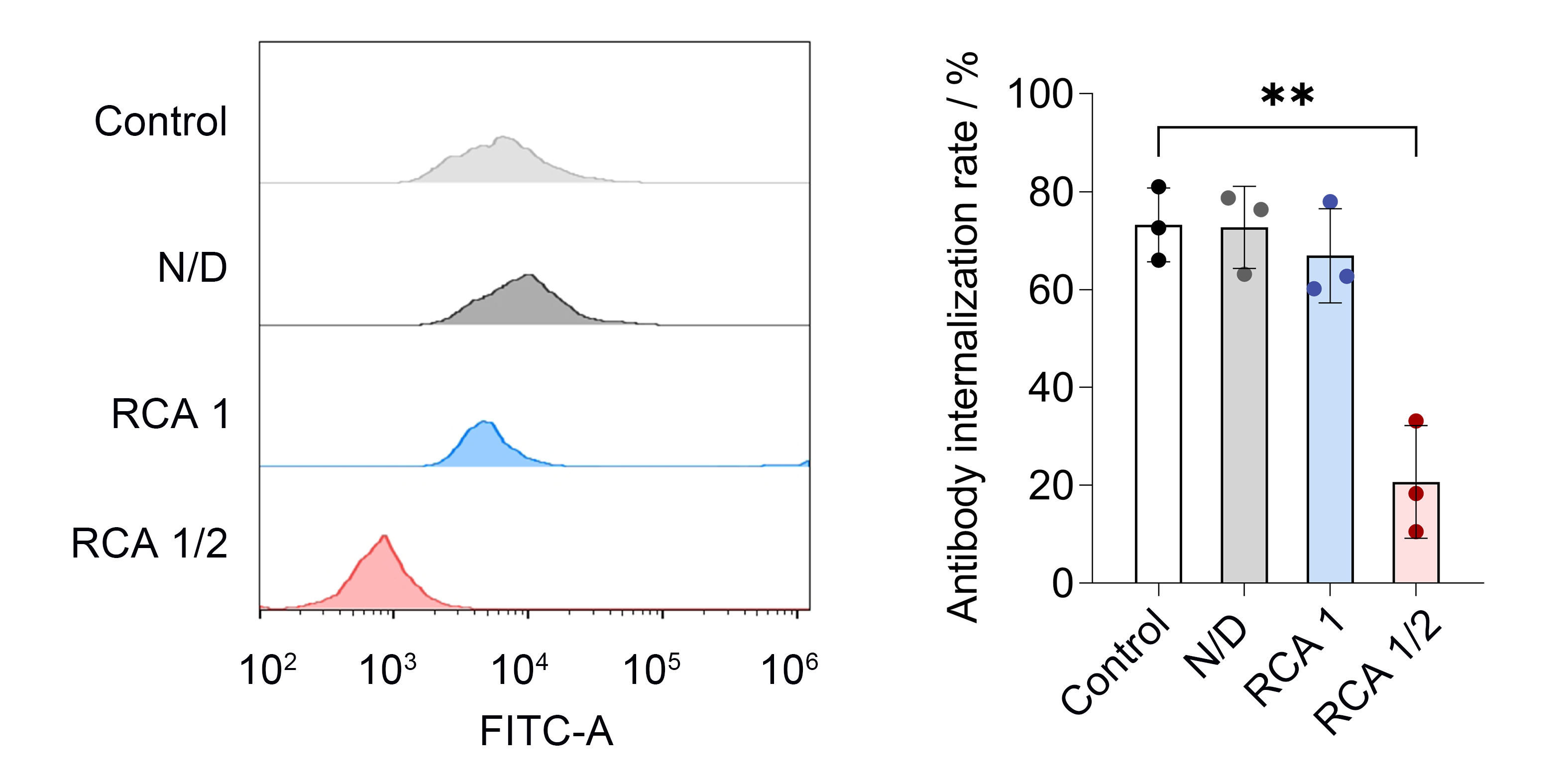


Figure S7 Anti-integrin antibody internalization assay. Cells (untreated control, D‑DNA‑engineered, RCA1 alone, and RCA1/2 network) were incubated with FITC-labeled anti-integrin antibody at 4 °C to label surface integrins, washed, shifted to 37 °C for 30 min to allow internalization, then treated with acid buffer to strip surface-bound antibody. Internalized fluorescence was quantified by flow cytometry. The RCA1/2 network group showed significantly reduced antibody internalization compared to the other three groups.Data are the means ± SD, *n*=3 independent experiments. *p* values were calculated by the Student’s *t*-test: **p* < 0.05, ***p* < 0.01, ****p* < 0.001.

Table S1. DNA sequences for experiments.

| Name | Sequence (5'→3') |
| --- | --- |
| D-DNA | DBCO-TTTTTTTTTTTTTTTTTTTTCAGAGTCAAGAGGAGTGAGT |
| Primer 1 | GCAGAGCGAAGTTTTTTTTACTCACTCC |
| CircDNA 1 | P-CTTCGCTCTGCACTGCATCGAAAAAAAACCCAACCCGCCCTAAAAAAAACAGAGTCAAGAGGAGTGAGTAAAAAAAA |
| Primer 2 | GCACTGCATCGTTTTTTTTACTCACTCC |
| CircDNA 2 | P-CGATGCAGTGCAGAGCGAAGAAAAAAAACACCCAAGCAGGCAAAAAAAACAGAGTCAAGAGGAGTGAGTAAAAAAAA |
| beacon 1, | Cy3-CGCTCTCCCAACCCGCCCTAGAGCG-BHQ2 |
| beacon 2 | Cy5-GCTCGCCACCCAAGCAGGCGCGAGC-BHQ2 |
| scrambled DNA | CTTCTTCTTCTTCTTCTTCTTCTTCTTCTTCTTCTTCTTC |

Table S2. Comparison of different cell surface engineering approaches.

| Approach | Efficiency | Reversibility | Biocompatibility | Mechanistic control |
| --- | --- | --- | --- | --- |
| Polymer coating [1] | High (70–75% cell binding) | No | Cells viable over 5 days, no toxicity reported | Surface chemistry (–NH₂/–COOH) which dictates cell adhesion and matrix remodeling |
| Lipid modification [2] | Not explored | No | 1  μM conjugates non‑toxic; 5  μM lipid‑peptide >70% viability | Lipid structure (chain length, cholesterol) dictates anchoring; cholesterol shows highest affinity |
| Protein-based system [3] | High (cargo transport and pattern formation on membranes) | Yes (ATP‑dependent; pattern can be dispersed by ATP depletion) | Lhysiologically benign, non‑cytotoxic | Diffusiophoretic transport; Turing pattern formation |
| This DNA self‑assembly network | High (83.08% cell binding) | Yes (using nuclease or strand displacement) | Low toxicity, no effect on viability/proliferation | Physical confinement: reduced membrane fluidity + integrin retention, independent of chemical signaling |

**Reference**

1. Pompe, T., Keller, K., Mothes, G., Nitschke, M., Teese, M., Zimmermann, R., & Werner, C. (2007). Surface modification of poly(hydroxybutyrate) films to control cell-matrix adhesion. Biomaterials, 28(1), 28–37. <https://doi.org/10.1016/j.biomaterials.2006.08.028>
2. Takahara, M., Wakabayashi, R., Fujimoto, N., Minamihata, K., Goto, M., & Kamiya, N. (2019). Enzymatic Cell-Surface Decoration with Proteins using Amphiphilic Lipid-Fused Peptide Substrates. Chemistry (Weinheim an der Bergstrasse, Germany), 25(30), 7315–7321. <https://doi.org/10.1002/chem.201900370>
3. Reverte-López, M., Gavrilovic, S., Merino-Salomón, A., Eto, H., Yagüe Relimpio, A., Rivas, G., & Schwille, P. (2023). Protein-Based Patterning to Spatially Functionalize Biomimetic Membranes. Small methods, 7(12), e2300173. <https://doi.org/10.1002/smtd.202300173>
